# Early epidemiological indicators, outcomes, and interventions of COVID-19 pandemic: A systematic review

**DOI:** 10.7189/jogh.10.020506

**Published:** 2020-12

**Authors:** Urvish Patel, Preeti Malik, Deep Mehta, Dhaivat Shah, Raveena Kelkar, Candida Pinto, Maria Suprun, Mandip Dhamoon, Nils Hennig, Henry Sacks

**Affiliations:** 1 Department of Public Health, Icahn School of Medicine at Mount Sinai, New York, USA; 2 Clinical Research Program, Icahn School of Medicine at Mount Sinai, New York, USA; 3 Department of Pediatrics, Allergy, and Immunology, Icahn School of Medicine at Mount Sinai, New York, USA; 4 Department of Neurology, Icahn School of Medicine at Mount Sinai, New York, USA; 5 Department of Environmental Medicine and Public Health, Pediatric Infectious Diseases, Health System Design and Global Health, Icahn School of Medicine at Mount Sinai, New York, USA; 6 Department of Environmental Medicine and Public Health, Icahn School of Medicine at Mount Sinai, New York, USA

## Abstract

**Background:**

Coronavirus disease-2019 (COVID-19), a pandemic that brought the whole world to a standstill, has led to financial and health care burden. We aimed to evaluate epidemiological characteristics, needs of resources, outcomes, and global burden of the disease.

**Methods:**

Systematic review was performed searching PubMed from December 1, 2019, to March 25, 2020, for full-text observational studies that described epidemiological characteristics, following MOOSE protocol. Global data were collected from the JHU-Corona Virus Resource Center, WHO-COVID-2019 situation reports, KFF.org, and Worldometers.info until March 31, 2020. The prevalence percentages were calculated. The global data were plotted in excel to calculate case fatality rate (CFR), predicted CFR, COVID-19 specific mortality rate, and doubling time for cases and deaths. CFR was predicted using Pearson correlation, regression models, and coefficient of determination.

**Results:**

From 21 studies of 2747 patients, 8.4% of patients died, 20.4% recovered, 15.4% were admitted to ICU and 14.9% required ventilation. COVID-19 was more prevalent in patients with hypertension (19.3%), smoking (11.3%), diabetes mellitus (10%), and cardiovascular diseases (7.4%). Common complications were pneumonia (82%), cardiac complications (26.4%), acute respiratory distress syndrome (15.7%), secondary infection (11.2%), and septic shock (4.3%). Though CFR and COVID-19 specific death rates are dynamic, they were consistently high for Italy, Spain, and Iran. Polynomial growth models were best fit for all countries for predicting CFR. Though many interventions have been implemented, stern measures like nationwide lockdown and school closure occurred after very high infection rates (>10cases per 100 000population) prevailed. Given the trend of government measures and decline of new cases in China and South Korea, most countries will reach the peak between April 1-20, if interventions are followed.

**Conclusions:**

A collective approach undertaken by a responsible government, wise strategy implementation and a receptive population may help contain the spread of COVID-19 outbreak. Close monitoring of predictive models of such indicators in the highly affected countries would help to evaluate the potential fatality if the second wave of pandemic occurs. The future studies should be focused on identifying accurate indicators to mitigate the effect of underestimation or overestimation of COVID-19 burden.

Since its inception in Wuhan, China in December 2019, coronavirus disease 2019 (COVID-19) has spread throughout the world and has been declared a pandemic by World Health Organization (WHO) on March 11, 2020 [1]. As of April 12020, there are 935 197 confirmed cases worldwide with 47 192 (5.04%) deaths and 193 989 (20.7%) recovered cases [2]. New York is the current epicenter of COVID-19 (102 863 cases and 2935 deaths) of United States of America (USA) (215 003 cases, 5102 (2.37%) deaths and 8878 (4.13%) recovered patients) while Italy (13 155 deaths) and Spain (10 003 deaths) being worst affected countries [2,3]. Globally, the epidemiological scenario of COVID-19 is changing on a daily basis.

The origin of severe acute respiratory syndrome coronavirus (SARS-CoV-2) virus was linked to a seafood market in Wuhan from the handling and close contact with animals [4]. In USA, the first case was reported on January 20, 2020, with a recent travel history to Wuhan [5]. According to emerging literature, COVID-19 symptoms can range from mild respiratory illness causing fever, dry cough, dyspnea, myalgia and fatigue to more severe manifestation of pneumonia, cardiac complications requiring intensive care unit (ICU) admission and mechanical ventilation [6]. The median incubation period is around 5 days (range:2-14 days), requiring prolonged monitoring in extreme cases [7,8]. Real-time reverse transcriptase polymerase chain reaction (RT-PCR) of nasopharyngeal and/or oropharyngeal swabs are usually used to confirm the diagnosis [9,10]. Preliminary demographic data of the infected patients suggests that most patients have mild disease, with older adults (≥65 years) appearing to be more susceptible to severe illness requiring hospitalization [11,12]. COVID-19 shows evidence of human to human transmission via respiratory droplets and from contact with contaminated surfaces or objects, with estimated median basic reproduction number (R0) of 2.28 (range: 2.06-2.52) [13], making the spread of the disease tough to contain.

While recently published observational studies have provided insights on the epidemiology of this pandemic, their sample sizes are too limited for any definitive conclusions. Hence, we sought to conduct a systematic review and analysis of all available studies comparing outcomes. Primary aim of the study is to evaluate the epidemiological characteristics, needs of resources, and patients’ outcomes. Secondary aim is to evaluate the global burden and interventions.

## METHODS

### Primary aim evaluation

#### Endpoints

We evaluated epidemiological characteristics, risk factors, laboratory and imaging findings, complications and treatment utilized. We also calculated the mortality, recovery, and needs of resources like ICU beds and mechanical ventilators.

#### Eligibility criteria, search strategy and selection criteria

In order to evaluate the primary outcome, we performed a systematic review of these observational studies according to MOOSE guidelines [13,14]. We searched the PubMed database for original observational studies that described any details on epidemiological characteristics on patients with COVID-19. The database was searched from December 1, 2019, to March 25, 2020. The search was conducted using the following keyword/MESH terms: ((COVID-19[Title/Abstract]) OR coronavirus [Title/Abstract]) OR SARS-CoV-2 [Title/Abstract] OR 2019-nCoV [Title/Abstract]. All studies that compared outcomes of interest in COVID-19 patients were included. Any literature other than observational studies was excluded. Non-English literature, non-full text, and animal studies were excluded. Abstracts were reviewed, and articles were retrieved accordingly. Two independent reviewers performed the search and literature screening (UP, PM), with disputes resolved by consensus following discussion with a third author (CP). For the ease of understanding, we used a flow diagram to describe literature search and study selection process in Figure S1 in the **Online Supplementary Document**.

#### Data collection

A prespecified data collection Excel sheet was used to collect the data relating to study characteristics and outcomes of interest by two authors (PM and CP), and discrepancies were solved by a discussion with a third author (UP). The following study characteristics were extracted: publication year, country of origin, sample size, age, sex, direct exposure to infection, travel history, signs and symptoms, risk factors and comorbidities, laboratory and radiology findings, treatment utilized, and complications. Data on the following outcomes were extracted: mortality, recovery, need for ICU beds and mechanical ventilators.

#### Statistical analysis

All analysis was done in Excel (Microsoft Inc, Seattle WA, USA) and SAS 9.4 (SAS Institute, Cary, NC, USA). The frequencies and percentages of epidemiological characteristics and outcomes were calculated.

### Secondary aim evaluation

We evaluated the global burden of COVID-19 including case fatality rates (CFR), strength of association between deaths and cases to predict CFR, case doubling time, COVID-19 specific mortality rates, and control measures by governments to prevent spread among USA, China, Italy, Iran, Spain, Germany, India, and South Korea. For this purpose, data were taken from the Johns Hopkins University Corona-Virus Resource Center [3], KFF.org [14], World Health Organization-COVID-2019 situation reports [15], and Worldometers.info [2] up until March 31, 2020. We evaluated changes in cases and deaths, CFR, created a predictive modeling for CFR, COVID-19 specific mortality rate, and doubling time for cases and deaths.

CFR was defined as the number of cases divided by the number of the diagnosed patients with COVID-19, and COVID-19 specific mortality rate was defined by deaths due to COVID-19 infections divided by total population of the country in 2020, counted per 100 000 population [16] Pearson correlation coefficient (r) was obtained to establish the strength of association between deaths and cases for individual countries. To predict CFR, we modelled the epidemic curves with simple linear regression, exponential growth, and polynomial growth models and used a coefficient of determination (R^2^) for model selection. The time of reporting the first death was used as the starting point for that country for all three models.

We utilized government websites, national media, and other standard open sources to evaluate the governments’ interventions during COVID-19 pandemic, infection rate [(diagnosed cases/country’s population in 2020) per 100 000 population] [16] at the time of interventions like nationwide school closure and lockdown, and effects of such measures to predict the dates of peak number of cases in each country.

## RESULTS

### Primary outcomes

Our search resulted in 1956 studies, out of which 1688 non-human studies and other than observational studies, 64 non-full text and articles with non-English language information were excluded. 224 full-text studies were screened and 45 studies with insufficient clinical information or outcomes-related information were excluded. 166 full-text articles were assessed for eligibility. The final analysis included 21 full-text observational studies, presented in **Table 1**, including a total of 2747 patients.

**Table 1:** Studies used for this systematic review

Author, month, year	Data collected in country	Dates of collected data	Confirmed case	Discharge/recovery	Deaths
Lauer, Mar 2020 [17]	China	Jan 4, 2020 – Feb 24, 2020	181	-	-
Huang, Jan 2020 [18]	China	Dec 16, 2019 – Jan 2, 2020	41	28	6
Guan, Feb 2020 [11]	China	Dec 11, 2019 – Jan 29, 2020	1099	64	15
Zhao, Mar 2020 [19]	China	Jan 23, 2020 – Feb 5, 2020	19	0	0
Young, Mar 2020 [20]	Singapore	Jan 23, 2020 – Feb 3, 2020	18	-	0
Chang, Feb 2020 [21]	China	Jan 16, 2020 – Jan 29, 2020	13	13	0
Wang, Feb 2020 [22]	China	Jan 1, 2020 – Jan 28, 2020	138	47	6
Ng, Mar 2020[23]	Singapore	Jan 2, 2020 – Feb 29, 2020	100	-	0
Spiteri, Mar 2020 [24]	Europe	Jan 24, 2020 – Feb 21, 2020	38	-	1
COVID-19 National Incident Room Surveillance Team, Mar 2020 [25]	Australia	7 Mar 2020	71	22	2
Xu, Feb 2020 [26]	China	Jan 10, 2020 – Jan 26, 2020.	62	-	0
Bajema, Feb 2020 [27]	USA	Jan 20, 2020	11	-	-
Ki, Feb 2020 [28]	South Korea	Jan 20, 2020	28	-	-
Chen, Jan 2020 [29]	China	Jan 1, 2020 – Jan 20, 2020	99	31	11
Zhang, Feb 2020 [30]	China	Jan 16, 2020 – Feb 3, 2020	140	-	-
Yang, Feb 2020 [31]	China	Dec 24, 2019 – Jan 26, 2020	52	8	32
Wang, Mar 2020 [32]	China	Jan 16, 2020 – Jan 29, 2020	69	18	5
Mo, Mar 2020 [33]	China	Jan 1, 2020 – Feb,1 2020	155	-	-
Arentz, Mar 2020 [34]	USA	Feb 20, 2020 – Mar 5, 2020	21	2	11
Wu, Mar 2020 [35]	China	Dec 25, 2019 – Jan 26, 2020.	201	-	44
Zhou, Mar 2020 [36]	China	Dec 29, 2019 – Jan 31, 2020	191	137	54

#### Epidemiological and clinical characteristics

A total of 2747 patients had a confirmed diagnosis of COVID-19 across these 21 studies, the majority of the data (14/21 of the studies) were from China. The mean age of the total study cohort was 48 ± 10.8(SD) years. 1599/2736 (58.4%) were male. 909/1929 (47.1%) patients had a history of direct exposure with the infected person, while 371/1540 (24.1%) had a recent history of travel to China. We found that most common clinical symptoms of COVID-19 patients were fever (2209/2438; 90.6%), cough (1656/2438; 67.9%), myalgia or fatigue (1179/2438; 48.3%), sputum production or expectoration (602/2438; 24.7%) and dyspnea (554/2438; 22.7%). Other clinical symptoms included headache (235/2438; 9.6%), sore throat (192/2438; 7.9%), nausea or vomiting (124/2438; 5.1%), diarrhea (121/2438; 4.9%), nasal congestion (25/2438; 1%) and hemoptysis (14/2438; 0.57%). Most common laboratory findings were increased C-reactive protein (CRP) levels (707/1388; 51%), increased lactate dehydrogenase (LDH) levels (700/1678; 41.7%), lymphocytopenia (634/1737; 36.5%), increased D-dimer levels (457/1590; 28.7%), and leukocytopenia (464/1668; 27.8%). Hypertension (422/2188; 19.3%), smoking (189/1678; 11.3%), diabetes mellitus (217/2169; 10%), and cardiovascular diseases (166/2244; 7.4%) were the most common coexisting comorbidities. 1321/1598 (82.7%) patients had abnormal chest computed tomography scan findings, with 1445/2197 (65.7%) patients had bilateral lungs affected, and 975/1637(59.5%) patients showed ground-glass opacity. 1406/2042 (68.8%), 1054/2144 (49.2%), 548/2107 (68.8%) and 275/1859 (14.8%) patients with confirmed COVID-19 were given antibiotics, antiviral medicines, corticosteroid and intravenous immunoglobulin as treatment respectively. Oxygen was administered in 1061/2063 (51.4%) patients. 42/1620 (2.6%) patients required continuous renal replacement therapy. Common complications of COVID-19 infection were pneumonia (1412/1722; 82%), cardiac complications (136/514; 26.4%), acute respiratory distress syndrome (291/1842; 15.7%), secondary infection (44/393; 11.2%), and septic shock (66/1527; 4.3%) **(****Table 2****).**

**Table 2:** Epidemiological, clinical, laboratory characteristics and outcomes of the patients infected with COVID-19 analyzed in this review

Total number of Confirmed Cases from Articles Reviewed	N = 2747
**Age (years) (Mean** **±** **SD)**	48 ± 10.3
**Gender:**	
Male	1599/2736 (58.4%)
Female	1130/2736 (41.3%)
Direct exposure with infected person	909/1929 (47.1%)
Travel history to China	371/1540 (24.1%)
China resident	623/1420 (43.8%)
**Symptoms:**	
Fever	2209/2438 (90.6%)
Cough	1656/2438 (67.9%)
Dyspnea	554/2438 (22.7%)
Myalgia or fatigue	1179/2438 (48.3%)
Sputum production/expectorant	602/2438 (24.7%)
Headache	235/2438 (9.6%)
Diarrhea	121/2438 (4.9%)
Nausea/vomiting	124/2438 (5.1%)
Hemoptysis	14/2438 (0.57%)
Sore throat	192/2438 (7.9%)
Nasal congestion	25/2438 (1%)
**Risk factors/comorbidities:**	
Hypertension	422/2188 (19.3%)
Smoking	189/1678 (11.3%)
Diabetes mellitus	217/2169 (10%)
Cardiovascular disease	166/2244 (7.4%)
Cerebrovascular disease	32/960 (3.3%)
Pulmonary disease	53/2169 (2.4%)
Chronic liver disease	53/2188 (2.4%)
Malignancy	39/2206 (1.8%)
Other comorbidities	380/2157 (17.6%)
**Laboratory parameters:**	
Leukopenia	464/1668 (27.8%)
Lymphopenia	634/1737 (36.5%)
Thrombocytopenia	340/1389 (24.5%)
Elevated aspartate transaminase (AST)	301/1527 (19.7%)
Elevated alanine transaminase (ALT)	316/1678 (18.8%)
Elevated c-reactive protein (CRP)	707/1388 (51%)
Elevated lactate dehydrogenase (LDH)	700/1678 (41.7%)
Elevated creatinine kinase	126/1408 (9%)
Elevated creatinine	32/1590 (2.01%)
Elevated D-dimer	457/1590 (28.7%)
Elevated total bilirubin	104/1399 (7.4%)
Elevated activated partial thromboplastin time (APTT)	25/292 (8.6%)
Elevated blood urea nitrogen (BUN)	6/99 (6%)
Elevated erythrocyte sedimentation rate (ESR)	55/224 (24.5%)
**Computed tomography (CT) scan use:**	
Abnormal CT chest	1321/1598 (82.7%)
Bilateral lung affected	1445/2197 (65.7%)
Ground glass opacity	975/1637 (59.5%)
**Treatments utilized:**	
Antibiotic use	1406/2042 (68.8%)
Antiviral use	1054/2144 (49.2%)
Corticosteroid use	548/2107 (26%)
Intravenous immunoglobulin use	275/1859 (14.8%)
Oxygen	1061/2063 (51.4%)
Continuous renal replacement therapy	42/1620 (2.6%)
**Complications:**	
Pneumonia	1412/1722 (82%)
Acute respiratory distress syndrome	291/1842 (15.7%)
Septic shock	66/1527 (4.3%)
Cardiac complication	136/514 (26.4%)
Secondary infection	44/393 (11.2%)
Other	210/1600 (13.1%)
**Outcomes:**	
Deaths	188/2243 (8.4%)
Recovery	370/1813 (20.4%)
Intensive care unit (ICU) admissions	322/2090 (15.4%)
Mechanical ventilation requirement	304/2035 (14.9%)

#### Patients’ outcomes

Overall, 188/2243 (8.4%) patients had died and 370/1813 (20.4%) patients had recovered. 322/2090 (15.4%) patients required ICU admission and 304/2035 (14.9%) patients required mechanical ventilation **(****Table 2****).**

### Secondary outcomes

#### Case fatality rate (CFR)

On January 22, 2020, CFR for China was 3.1% which remained between 2%-4% until March 31. On March 4, CFR for USA was 7.38% which declined to 2.06% on March 31. Initially in Italy, on February 21 CFR was 5%, reduced to 2.01% on March 1, then increasing to 11.7% on March 31. Spain’s CFR was 0.61% on March 3 which increased to 8.82% on March 31. For South Korea, CFR was 0.96% on February 20, slowly reaching 1.66% on March 31. CFR in India was 1.61% on March 11, which has increased to 2.51% on March 31. Germany had a CFR of 0.17% on March 9 which increased to 1.08% on March 31. In Iran, on Feb 19 CFR was almost 100% which reduced to 3.3% on March 3 with larger numbers of cases and from then it has increased and reached 6.5% on March 31. The changes in the country-specific CFR are plotted in Figure S2 in the **Online Supplementary Document****.**

#### Strength of association between deaths and cases to predict CFR (Predictive modeling)

**Figure 1:**
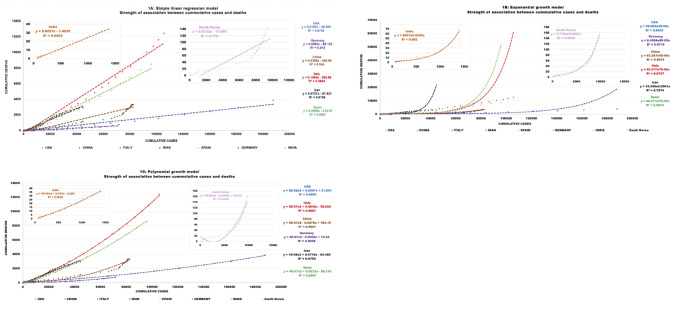
Modeling to determine the type of association between cumulative deaths and cumulative cases to predict case fatality rate (CFR). **Panel A.** Simple linear regression. **Panel B.** Exponential growth model. **Panel C.** Polynomial (quadratic) growth model.

Several models, including a simple linear regression, exponential and polynomial (quadratic) growth models, were used to determine the type of association between cumulative deaths and cumulative cases to predict CFR (**Table 3**). The polynomial growth model had the best fit (higher R^2^) and indicates that for all countries the death rate increases with the number of cases, and this increase is steeper than a linear relationship. Interestingly, while for the USA, Italy, Iran, Spain, and India this association is always positive, for China, South Korea, and Germany the initial slope is negative but then is reversed as the number of cases continues to increase **(****Figure 1****).**

**Table 3:** Country-specific strength of association between deaths and cases (predicted case fatality rate, CFR)

Country	Pearson correlation (r)	Simple linear regression (SLR) model to evaluate relationship between cumulative deaths (y) and diagnosed cases (x) [CFR = y/x]	Coefficient of determination (R^2^) for SLR model	Exponential growth (EG) model to evaluate relationship between cumulative deaths (y) and diagnosed cases (x), [CFR = y/x]	Coefficient of determination (R^2^) for EG model	Polynomial growth (PG) model to evaluate relationship between cumulative deaths (y) and diagnosed cases (x) [CFR = y/x]	Coefficient of determination (R^2^) for PG model
USA	0.99	y = 0.0182x - 48.4	0.979	y = 29.002e^0.00003x^	0.663	y = 0.00000006x^2^ + 0.0097x + 21.651	0.999
Germany	0.96	y = 0.0093x - 68.1	0.915	y = 6.4584e^0.00008x^	0.872	y = 0.0000002x^2^ - 0.0006 + 10.24	0.996
China	0.96	y = 0.0399x - 309	0.924	y = 83.267e^0.00005x^	0.907	y = 0.0000005x^2^ - 0.0079x + 183.16	0.969
Italy	0.99	y = 0.1094x - 399.7	0.986	y = 59.277e^0.00007x^	0.673	y = 0.00000057x^2^ + 0.0642x - 58.624	0.999
Iran	0.99	y = 0.0721x - 87.4	0.976	y = 33.548e^0.0001x^	0.727	y = 0.00000001x^2^ + 0.0716x - 85.485	0.976
Spain	0.99	y = 0.0859x - 317	0.987	y = 40.211e^0.00007x^	0.668	y = 0.0000004x^2^ + 0.0533x - 88.124	0.999
South Korea	0.88	y = 0.0133x - 18	0.779	y = 3.7164e^0.0004x^	0.923	y = 0.000003x^2^ - 0.0148x + 18.415	0.945
India	0.99	y = 0.0257x - 1.4	0.980	y = 1.8001e^0.0026x^	0.892	y = 0.000005x^2^ + 0.019x - 0.285	0.985

#### COVID-19 specific death rate

COVID-19 specific death rates (per 100 000 population) for individual countries are presented in Figure S3 in the **Online Supplementary Document****.** In USA, cumulative COVID-19 specific death rate is steadily increasing and has reached 1.17, lower than in other countries like Italy (20.55 : exponentially increasing), Iran (3.45:constantly increasing), and Spain (16.10:exponentially increasing) but higher than China (0.23:almost steady), Germany (0.925:constantly increasing), South Korea (0.316:slowly increasing) and India (0.002:constantly increasing) as of April 1 (Figure S3A in the **Online Supplementary Document**). The daily COVID-19 specific death rate is highest in Spain (daily 1.6 deaths per 100 000 population) and Italy (daily 1.38 deaths per 100 000 population) followed by USA (daily 0.27 deaths per 100 000 population) (Figure S3B in the **Online Supplementary Document**).

#### Doubling time

The county-specific timeline of doubling time for cases and deaths is shown in **Table 4** and the increment in cases and deaths are plotted in (Figure 4 in the **Online Supplementary Document**).

**Table 4:** Country specific doubling time for cases and deaths

Dates	Doubling time for cases (days)	Dates	Doubling time for deaths (days)
**USA [First case reported: January 22, 2020; First death reported: February 29, 2020]:**			
January 22-January 26	2	February 29-March 2	2
January 26-February 3	8	March 2-March 5	3
February 3-February 24	21	March 5- March 15	5
February 24-March 2	7	March 15-March 18	3
March 2-March 30	2-4	March 18-March 29	2-3
**China [First case reported: December 31, 2019; First death reported: January 11, 2020]:**			
January 22 – January 25*	3	January 22 – February 2*	2-3
January 25 – January 29	2	February 2 – February 6	4
January 29 – February 4	3	February 6 – February 13	7
February 4 – February 13	9	February 13 – February 26	13
February 13 – March 31†	>47	February 26 – March 31†	>34
**Italy [First case reported: January 31, 2020; First death reported: February 21, 2020]:**			
January 31 – February 21	21	February 21 – February 24	1-2
February 21 – February 29	1-2	February 24 – March 15	2-4
February 29 – March 15	3-4	March 15 – March 20	5
March 15 – March 20	5	March 20 – March 26	6
March 20 – March 29	9	March 26 – March 31^†^	>5
**Iran [First case reported: February 19, 2020; First death reported: February 19, 2020]:**			
February 19 – March 2	1-2	February 19 – February 25	2
March 2 – March 5	3	February 25 – February 28	3
March 5 – March 9	4	February 28 – March 15	4
March 9 – March 16	7	March 15 – March 21	6
March 16 – March 27	11	March 21 – March 31†	>10
**Spain [First case reported: February 1, 2020; First death reported: March 3, 2020]:**			
February 1 – February 9	8	March 3 – March 15	1-2
February 9 – February 25	16	March 15 – March 24	3
February 25 – March 13	1-3	March 24 – March 28	4
March 13 – March 31	4-6	March 28 – March 31†	>3
**Germany [First case reported: January 27, 2020; First death reported: March 9, 2020]:**			
January 27 – February 1	1-4	March 9 – March 13	4
February 1 – February 11	10	March 13 – March 28	3
February 11 – February 27	16	March 28 – March 31†	>3
February 27 – March 19	2-4		
March 19 – March 29	5		
**South Korea [First case reported: January 20, 2020; First death reported: February 20, 2020]:**			
January 22 – January 31	1-4	February 20 – February 21	1
January 31 – February 6	6	February 20 – February 26	2-3
February 6 – February 20	14	February 26 – March 2	5
February 20 – March 1	1-3	March 2 – March 11	9
March 1 – March 9	8	March 11 – March 24	13
March 9 – March 31†	>21	March 24 – March 31†	>7
**India [First case reported: January 30, 2020; First death reported: March 11, 2020]:**			
January 30 – February 2	3	March 11 – March 13	2
February 2 – March 2	29	March 13 – March 19	6
March 2 – March 4	2	March 19 – March 23	4
March 4 – March 20	5-6	March 23 – March 26	3
March 20 – March 23	3	March 26 – March 31†	>5
March 23 – March 29	6		

*Data on daily cases and deaths are unavailable before January 22, 2020.

†Deaths or cases have not yet doubled from the previous respective dates.

#### Interventions

**Table 5** summarizes the different interventions adopted by different countries in an effort to mitigate the spread of the COVID-19 virus.

**Table 5:** Measures taken by various countries to prevent spread and mitigate the risk

United States of America (USA)	
29 January	White House Coronavirus Task Force established to monitor, prevent, contain, and mitigate the spread of the pandemic in the USA [37]
31 January	Declared public health emergency[38]; Travel restrictions placed on entry from China [38]
6 March	The President signed the Coronavirus Preparedness and Response Supplemental Appropriations Act (CPRSA) providing $8.3 billion in emergency funding for federal agencies to respond to the outbreak [39]
12 March	All 50 states are permitted to perform tests by a doctor's approval, from the CDC or commercial laboratories[40] and appointment of Admiral Brett Giroir of the U.S Public Health Service to oversee testing, funding for two companies developing rapid tests, and a hotline to help laboratories find needed supplies [41]
13 March	National emergency was declared, making federal funds available to respond to the crisis [42], Drive-through testing began [43]
16 March	President Trump’s Coronavirus guidelines for America [44]
19 March	Lockdown in California [45]
20 March	Barred entry of foreign nationals who had been to 28 European countries within last 14 days [46]
22 March	Nationwide schools closed [47], Lockdown in New York [45]
27 March	A US$ 2 trillion coronavirus stimulus bill was passed and signed by the President [48]
30 March	More than half of US states underwent lockdown [45]
**China:**	
22 January	Response to Public Health Emergency launched by Hubei [49]
23 January	The Central government of China imposed a lockdown in Wuhan and other cities in Hubei province; Public transport suspended. The Wuhan airport, railway stations and metro were closed, not allowing residents to leave the city without permission [50]; Public Health Emergency response announced by mainland province of Zhejiang [51]
29 January	Mainland China has initiated Public Health Emergency response [52]; Quarantined whole Hubei Province [53]; Curfew laws implemented in Huanggang,Wenzhou and other mainland cities [54]
**South Korea:**	
4 February	An unlicensed Covid-19 test authorized by the Korea Centers for Disease Control and Prevention (CDC) [55]; Travel denied to foreign nationals from Hubei Province into South Korea [56]
23 February	All kindergartens, elementary schools, middle schools, and high schools were announced to delay the semester start [57]
26 February	Entire country opened drive-through testing [58]
**Italy:**	
31 January	State of emergency declared, flights to and from China suspended [59]
22 February	The Council of Ministers announced a new decree-law to quarantining more than 50 000 people from 11 different municipalities in Northern Italy [60]
4 March	Nationwide schools and universities closed [61]
10 March	Prime Minister imposed Nationwide quarantine lockdown [62]
11 March	All commercial activities except pharmacies and supermarkets ordered to shut down [63]; €25billion allocated by the government [64]
1 April	Drive-through testing began [65]
**Iran:**	
22 February	All concerts and other cultural events cancelled for one week by Ministry of Islamic Culture and Guidance [66]; Closure of educational institutions in several cities and provinces announced by the Ministry of Health and medical education [67]
5 March	Checkpoints placed between cities to limit travel [68]
16 March	Fatima Masumeh Shrine, Jamkaran Mosque in Qom city, and Imam Reza Shrine in Mashhad closed [69]
**Germany:**	
28 February	New health security measures enacted to regulate air and sea travel that required passengers from China, South Korea, Japan, Italy and Iran to report their health status before entry [70]; Federal police stepped up checks within 30 km of the border [70]
16 March	Bavaria declared a state of emergency for 14 days and measures to limit public movement and additional funds for medicine supplies were introduced [71]; All flights from Iran and China stopped by German Ministry of Transport [72]; Travelling in coaches, attending religious meetings, visiting playgrounds or engaging in tourism prohibited [73]
17 March	Immediate travel ban into the European Union for 30 days for non-EU citizens was announced by Merker[74] followed by widening travel restrictions to EU citizens from Italy, Switzerland, Denmark, Luxembourg and Spain [75]
20 March	Bavaria became the first state to declare a curfew [76]
22 March	Gathering of more than 2 people forbidden by the government for at least 2 weeks with a minimum distance of 1.5 m between people in public [77]
**Spain:**	
10 March	All direct flights from Italy to Spain cancelled [78]
13 March	Bars, restaurants and “non-alimentary” shops ordered to shut down by Government of the Community of Madrid [79]
15 March	Nationwide lockdown imposed by the Spanish government [80]
16 March	Spanish government announced the closing of its land borders [81]
**India:**	
3 March	Issuing of new visas and visas already issued for nationals of Italy, Iran, South Korea, and Japan suspended by the Indian government [82]
4 March	Compulsory screening of all international passengers arriving in India announced by the Minister of Health and Family Welfare [83]
11 March	All visas to India suspended by the government; All Indian nationals coming from COVID-19 hit nations after 15 February needed to be quarantined for 14 days [82]
15 March	65 laboratories of the Department of Health Research and the Indian Council of Medical Research (DHR-ICMR) started testing samples of people who exhibited flu-like symptoms and samples from patients without any travel history or contact with infected persons [84]
17 March	The Government of India issued an advisory urging all states to take social distancing measures as a preventive strategy [82]
22 March	Janata curfew (people's curfew) imposed [82]
23 March	Use of hydroxychloroquine for treatment of COVID19 for high-risk cases recommended by National Task Force constituted by ICMR [85]
25 March	Nationwide lockdown for 21 days [86]; Indian airspace closed [87]
26 March	Finance minister announced US$24 billion stimulus package [88]

**Infection rate at the beginning of the major intervention (nationwide closure of school or major lockdown):** Infection rate (number of diagnosed cases per 100 000 population) for the USA (March 22: 10.04; March 30: 48.81) was higher than China (January 23: 0.42), South Korea (February 23: 1.17) and India (March 25: 0.048). Infection rates were higher in Italy (March 10: 20.61), Iran (March 16: 17.85), Spain (March 14: 13.67), and Germany (March 22: 29.69).

**Travel restrictions:** China (January 23: Wuhan public transport) followed by Italy (January 31: all flights) and USA (January 31: flights from China; March 20: flights from European Union**),** South Korea (February 4: Hubei Foreigners), Iran (March 5: checkpoints between cities), Spain (March 10: all flights; March 16: closure of land borders), Germany (March 16: flights from China and Iran; March 17: European Union), India (March 4: mandatory screening of international passengers; March 11: suspension of all visas; March 25: closing of airspace).

**Quarantines, lockdowns, and social distancing measures:** China (January 29: Hubei, some cities of China) followed by Northern Italy (February 22). Lockdown measures were put in China (January 23: Wuhan, other cities of Hubei), Italy (March 10), Spain (March 14), California (March 19), Bavaria-Germany (March 20), New York (March 22) and India (March 25). Nationwide educational institutes closure: Iran (February 22), Italy (March 4) and USA (March 22). All social and religious gathering canceled: Iran (February 22: concerts and cultural events; March 16: mosques and shrines), Germany (March 16; March 22: no gathering of >2 people).

**Other actions:** China: public health emergency response (January 22: Hubei; January 29:mainland China); Italy: state of emergency (January 31); USA: White House Coronavirus Task Force (January 29), public health emergency (January 31), US$ 8.3 billion emergency funding (March 6), national emergency (March 13), $2 trillion stimulus bill (March 27); Spain: state of alarm (March 14); Bavaria-Germany: state of emergency (March 16); India: US$ 24 billion stimulus package (March 26).

**Testing:** The drive-through centers are operational in South Korea from February 26, USA from March 13, and Italy from April 1. As of April 1, cumulative tests conducted (per million population) in the USA (3470) are higher than Iran (952), China (222), India (35) and lower than Germany (11 127), Italy (9156), South Korea (8184), Spain (7593). The tests conducted (per day) in the USA (100 989) are higher than Italy (34 455), South Korea (10 983), India (5163) and Germany (5902) [89,90].

#### Impact of strict interventions on predicted days to reach peak number of diagnosed cases

**Table 6** mentions the predicted dates of the peak number of cases based on strict interventions. In China and South Korea, it took 16-21 days and 11-14 days respectively in order to achieve the peak of the pandemic before the new number of cases began to decline. We have used a 16-21 days post-interventional model to calculate the peak of the pandemic keeping in mind the effect of China's model of interventions.

**Table 6:** Country-specific predicted dates of peak numbers of cases according to strict interventions

Country	Strict interventions (Lockdowns and closure of educational institutes)	Days to reach peak (days)	Peak number of cases*
China	January 23-January 29	16-21	February 13-February14
South Korea	February 18-February 23	11-14	February 29-March 3
Predicted dates of peak numbers of cases:*			
Italy	March 4-March 11	16-21	March 20-April 1
Iran	March 16	16-21	April 1-April 6
Spain	March 14	16-21	March 30-April 4
Germany	March 22	16-21	April 7-April 12
USA	March 22-March 30	16-21	April 7-April 20
India	March 25	16-21	April 10-April 15

*Predicted dates based on implementation of strict lockdown of non-essential businesses, school, and public gathering. We have taken 16-21 days, keeping in mind the effect of China's model of interventions.

## DISCUSSION

COVID-19 has significantly impacted the entire world both socially and economically. The rapid human-to-human transmission has posed a great public health threat. Across 21 studies included in this review, we found 2747 confirmed cases of COVID-19 with the majority of the published studies from China. 47% of the cases had a history of direct exposure or being exposed to the seafood market in Wuhan, 44% were China residents and 24% had a travel history to China. Initially the virus was limited to only Wuhan and despite travel restriction, the virus continued to spread across the world at a rapid rate from China, likely due to asymptomatic transmission in the initial stages of the outbreak with a median incubation period of only 5 days [7,17], before travel restrictions. The COVID-19 cases are increasing exponentially but underestimated due to mild symptoms in a portion of cases, long incubation periods, and shortage of testing kits. In concurrence with other studies [18,29], we found that clinical characteristics of COVID-19 are similar to those of SARS and influenza virus. Fever (91%), cough (68%) and myalgia or fatigue (48%) were the most prominent symptoms. 24% of patients reported dyspnea and sputum production/expectoration. Major comorbidities were hypertension, smoking, diabetes mellitus, and cardiovascular disease. Patients with these comorbidities are at high risk for complications including pneumonia, ARDS and cardiovascular complications. We found that patients had increased inflammatory markers including elevated CRP in 50%, lymphopenia in 36% and elevated ESR in 25% which is similar to other respiratory infections (SARS, influenza). Few studies [18,91], have reported abnormal liver function in COVID-19 patients, and we found 20% of patients had elevated ALT and AST. Additionally, increased LDH (42%), D-dimer(29%) may indicate the severity of the disease[92]. Some studies have also reported elevated neutrophil count and cytokine storm induced by virus leading to coagulation activation and sustained inflammatory response [22] associated with higher mortality [29].

There is no proven therapy available as of now for COVID-19. Few studies like Wang et al. showed that remdesvir and hydroxychloroquine have in vitro efficacy in inhibiting the SARS-CoV-2 virus [93], Gao et al. suggest higher efficacy of hydroxychloroquine compared to supportive treatment [94] and Gautret et al. suggest enhanced efficacy with azithromycin and hydroxychloroquine combination [95]. Large scale clinical trials for these drugs are under way. 50% patients received oxygen and antibiotics (69%), antivirals (49%) and steroids (26%) as supportive therapies. The prognosis of patients after receiving these treatments is not yet clear. In people with compromised immune systems such as older age, HIV, malignancy, diabetes, chronic pulmonary disease if treated promptly with antibiotics, convalescent plasma to increase the immune support might reduce the risk of complications and mortality [96].

In our analysis, 15% of the patients required ICU admission, 15% needed mechanical ventilation, 8% died and 20% recovered and were discharged from the hospital. These findings are consistent with Guan et al. and Wang et al that present similar rates [11,22]. Currently in the USA, COVID-19 is in the acceleration phase surpassing China and Italy, and a National emergency was declared by the President, but the duration and severity may vary depending on the virus characteristics and public health response [97]. If confirmed cases continue to grow with this trend, soon the COVID-19 pandemic will cause shortages of ventilators. As per Institute for Health Metrics and Evaluation (IHME) projections, on a peak day in the USA, there would be a shortage of ICU beds by 19 863 and a need of 31 782 ventilators [98]. The growing number of cases will place a burden on the current capacity of hospitals and hence it is essential to develop and implement strategies to mitigate the gap by increasing capacity and fair allocation of available resources.

As of March 31, CFR in Italy was 11.75% and 4.01% in China. According to Onder et al. [99], CFR stratification by age, shows similar rates for 0-60 years (0%-3.6%) but higher in >70 years(8%-20.2%).This difference might be due to high CFR reported in people >90 years in Italy and no data from China for the same age group [99]. Other reasons might be demographics differences between two countries (≥65 years population: Italy-22.8% vs China-10.9%), overwhelming health care system, and shortage of ICU beds and ventilators, which might lead to prioritizing treatment to younger and otherwise healthy patients over older patient [100]. In our analysis CFR in Italy increased from 1.94% on February 23 to 8.57% on March 20, possibly due to the implementation of a strict policy of testing only suspected cases with severe symptoms [99]. Though widespread and drive-through testing is becoming more available in USA, cumulative tests conducted per million population lags behind compared to Germany, Italy, South Korea, and Spain. Our data driven polynomial growth model predicts more deaths in future with an increase in cases in USA [98], Italy, Iran, Spain, and India. As per our model predictions, doubling time of cases in the USA, Germany and India is decreasing suggesting that they are inching towards the peak. Different countries undertook interventions at different points in the timeline of spread of virus. The infection rates in the USA, Italy, Iran, Spain, and Germany were higher when they undertook substantial measures compared to China, South Korea, and India, suggesting a delayed response and failure to undertake timely measures. The aforementioned timelines for peaks look optimistic because multiple other factors may influence the trajectory of spread, ie, population density, economy, demographics, health care, religious beliefs, and legislation. For instance, despite the growing number of cases, Iran continued to keep its shrines open to pilgrims for a long time, but recently closed them, and no stringent curfew laws were imposed. Also, many states in USA have still not implemented strict quarantine measures. Such practices can seriously impede the efforts at containing the spread and skew the projection in many ways. Restrictions have neither been homogeneously imposed nor simultaneously adopted throughout the country, making it difficult to predict the exact model of the spread.

Also, COVID-19 testing capacity of the nations are limited and the true number of the infected people might have been higher than the estimated numbers at the time of our analysis. Hence, an early phase COVID- 19 specific death rate would be a better estimate than CFR to compare the severity of the disease. Many factors contribute to the accurate estimation of CFR such as testing capacity, care seeking and lack of understanding of the proportion of asymptomatic and pre symptomatic cases [101,102]. Limited knowledge of these factors in the early COVID-19 phase might have contributed to overestimation of CFR in our study. The use of serological testing for presence of IgM or IgG antibodies against SARS-CoV-2 will provide a better estimate of cumulative prevalence of COVID 19 infection [103]. As recommended by WHO, measuring the seroprevalence of antibodies to COVID-19 is crucial and will contribute to determine accurate CFR and help plan adequate public health response [104].

### Limitations, strengths, and future directions

The research on COVID-19 is rapidly evolving and new publications are becoming available daily. The majority of the epidemiologic data are coming from single center with limited sample sizes. To overcome this limitation and provide a global view of the COVID-19 pandemic, we have analyzed data on over 2500 patients from 21 peer-reviewed studies. As a result, we provided more generalizable estimates of laboratory findings, clinical symptoms and complications of COVID-19 patients. We have included data from several countries/regions; however, one limitation is that the majority of cohorts are from China, and as more data from other countries become available, additional meta-analyses would be essential. This is the first study rigorously tracking the timing of government interventions across multiple countries; however, as mentioned earlier, the adherence to those interventions could vary from one country to another, making the projections of the potential effectiveness challenging. We have not evaluated the duration of strict interventions in all these countries. The population prevalence data are based on the symptomatic patients with confirmed RT-PCR testing. Since some patients can be infected and present mild or no symptoms, or have not undergone RT-PCR testing, serological antibody testing in the future may allow a more accurate understanding of the disease prevalence and death rates. Despite all the limitations, this is the first study in our knowledge, highlighting and explaining epidemiological indicators, testing capacity, interventions, and expected burden of the COVID 19 at early phase.

## CONCLUSIONS

We have reviewed the burden of this pandemic and steps taken by the governments of different countries. Though the governments can continue strict lockdowns, it is not a long-term solution. Good hand hygiene, widespread testing, detection and isolation of new cases, rigorous contact tracing in low-prevalence settings, early vaccine development and its quick distribution, strengthening the overburdened health care system, and protecting frontline health care workers may help to gradually relax the strict lockdowns and cope with COVID-19 pandemic. This would only be possible by a collective approach undertaken by responsible governments, wise strategy implementation, and receptive populations. The future studies should be focused on identifying accurate indicators to mitigate the effect of underestimation or overestimation of COVID-19 burden. Close monitoring of such indicators in highly affected countries is very crucial to evaluate the potential fatality if the second wave of pandemic occurs.

## Additional material

Online Supplementary Document
