# Supplementary material for: Early epidemiological indicators, outcomes, and interventions of COVID-19 pandemic: A systematic review
Source: J Glob Health. 2020 Aug 15;10(2):020506. doi: 10.7189/jogh.10.020506 (PMC7567430; doi:10.7189/jogh.10.020506)
Supplement: Online Supplementary Document [file jogh-10-020506-s001.pdf]

Early epidemiological indicators, outcomes, and interventions of COVID-19 pandemic: A systematic review

Figure S1: Flow diagram of literature search and selection process of COVID-19
Figure S2: Country-specific Case Fatality Rate (CFR)
Figure S3: COVID-19 Specific death rate
Figure S3A: COVID-19 Specific death rate (Cumulative-change)
Figure S3B: COVID-19 Specific death rate (Daily-change)
Figure S4: County-specific timeline of doubling time for cases and deaths

Figure S1: Flow diagram of literature search and selection process of COVID-19

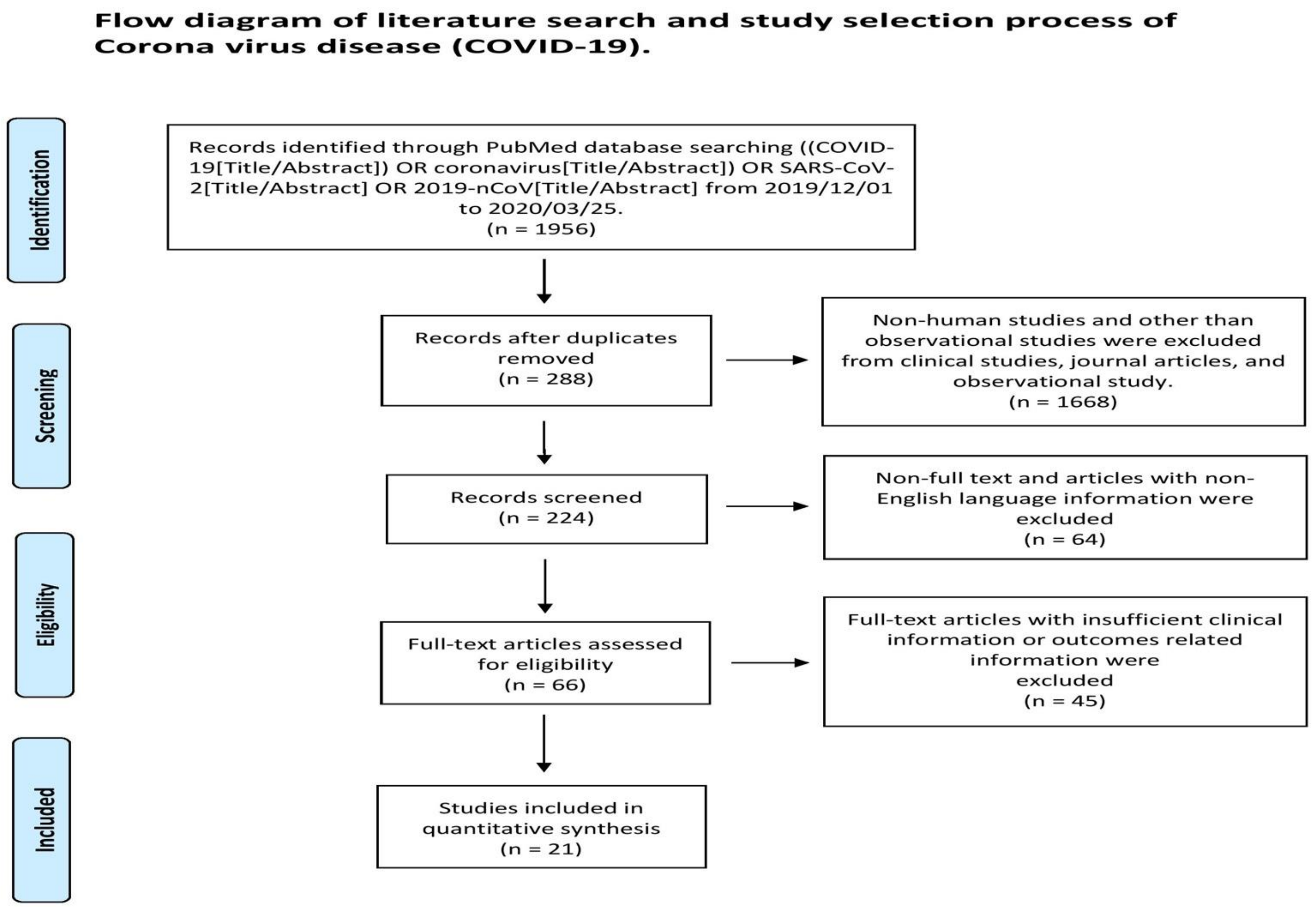

Figure S2: Country-specific Case Fatality Rate (CFR)

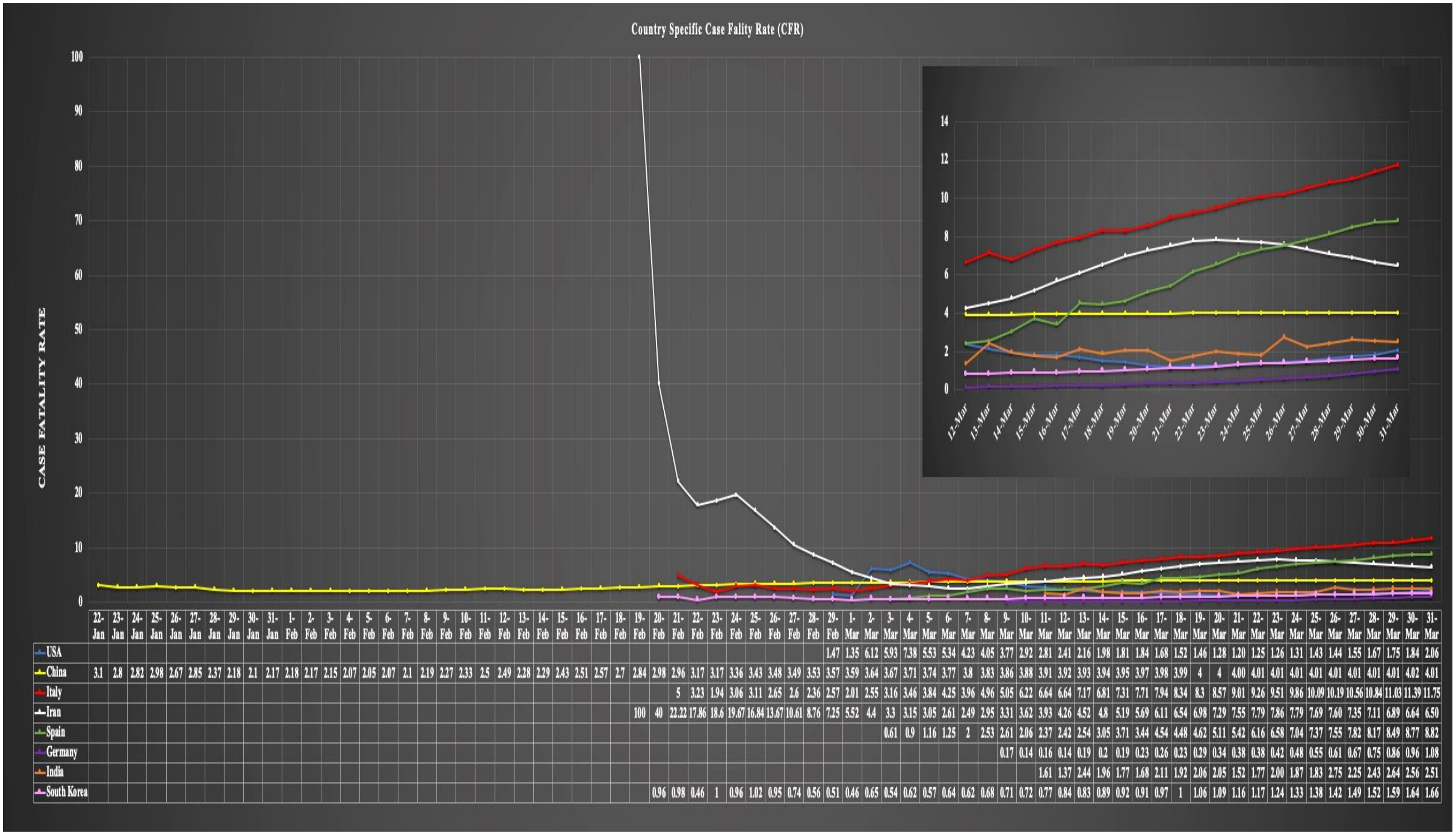

	22-Jan	23-Jan	24-Jan	25-Jan	26-Jan	27-Jan	28-Jan	29-Jan	30-Jan	31-Jan	1-Feb	2-Feb	3-Feb	4-Feb	5-Feb	6-Feb	7-Feb	8-Feb	9-Feb	10-Feb	11-Feb	12-Feb	13-Feb	14-Feb	15-Feb	16-Feb	17-Feb	18-Feb	19-Feb
USA																													
China	3.1	2.8	2.82	2.98	2.67	2.85	2.37	2.18	2.1	2.17	2.18	2.17	2.15	2.07	2.05	2.07	2.1	2.19	2.27	2.33	2.5	2.49	2.28	2.29	2.43	2.51	2.57	2.7	2.84
Italy																													
Iran																													100
Spain																													
Germany																													
India																													
South Korea																													

	20-Feb	21-Feb	22-Feb	23-Feb	24-Feb	25-Feb	26-Feb	27-Feb	28-Feb	29-Feb	1-Mar	2-Mar	3-Mar	4-Mar	5-Mar	6-Mar	7-Mar	8-Mar	9-Mar
USA										1.47	1.35	6.12	5.93	7.38	5.53	5.34	4.23	4.05	3.77
China	2.98	2.96	3.17	3.17	3.36	3.43	3.48	3.49	3.53	3.57	3.59	3.64	3.67	3.71	3.74	3.77	3.8	3.83	3.86
Italy		5	3.23	1.94	3.06	3.11	2.65	2.6	2.36	2.57	2.01	2.55	3.16	3.46	3.84	4.25	3.96	4.96	5.05
Iran	40	22.22	17.86	18.6	19.67	16.84	13.67	10.61	8.76	7.25	5.52	4.4	3.3	3.15	3.05	2.61	2.49	2.95	3.31
Spain													0.61	0.9	1.16	1.25	2	2.53	2.61
Germany																			0.17
India																			
South Korea	0.96	0.98	0.46	1	0.96	1.02	0.95	0.74	0.56	0.51	0.46	0.65	0.54	0.62	0.57	0.64	0.62	0.68	0.71

	10-Mar	11-Mar	12-Mar	13-Mar	14-Mar	15-Mar	16-Mar	17-Mar	18-Mar	19-Mar	20-Mar	21-Mar	22-Mar	23-Mar	24-Mar	25-Mar	26-Mar	27-Mar	28-Mar	29-Mar	30-Mar	31-Mar
USA	2.92	2.81	2.41	2.16	1.98	1.81	1.84	1.68	1.52	1.46	1.28	1.20	1.25	1.26	1.31	1.43	1.44	1.55	1.67	1.75	1.84	2.06
China	3.88	3.91	3.92	3.93	3.94	3.95	3.97	3.98	3.99	4	4	4.00	4.01	4.01	4.01	4.01	4.01	4.01	4.01	4.01	4.02	4.01
Italy	6.22	6.64	6.64	7.17	6.81	7.31	7.71	7.94	8.34	8.3	8.57	9.01	9.26	9.51	9.86	10.09	10.19	10.56	10.84	11.03	11.39	11.75
Iran	3.62	3.93	4.26	4.52	4.8	5.19	5.69	6.11	6.54	6.98	7.29	7.55	7.79	7.86	7.79	7.69	7.60	7.35	7.11	6.89	6.64	6.50
Spain	2.06	2.37	2.42	2.54	3.05	3.71	3.44	4.54	4.48	4.62	5.11	5.42	6.16	6.58	7.04	7.37	7.55	7.82	8.17	8.49	8.77	8.82
Germany	0.14	0.16	0.14	0.19	0.2	0.19	0.23	0.26	0.23	0.29	0.34	0.38	0.38	0.42	0.48	0.55	0.61	0.67	0.75	0.86	0.96	1.08
India		1.61	1.37	2.44	1.96	1.77	1.68	2.11	1.92	2.06	2.05	1.52	1.77	2.00	1.87	1.83	2.75	2.25	2.43	2.64	2.56	2.51
South Korea	0.72	0.77	0.84	0.83	0.89	0.92	0.91	0.97	1	1.06	1.09	1.16	1.17	1.24	1.33	1.38	1.42	1.49	1.52	1.59	1.64	1.66

Figure S3: COVID-19 Specific death rate

Figure S3A: COVID-19 Specific death rate (Cumulative-change)

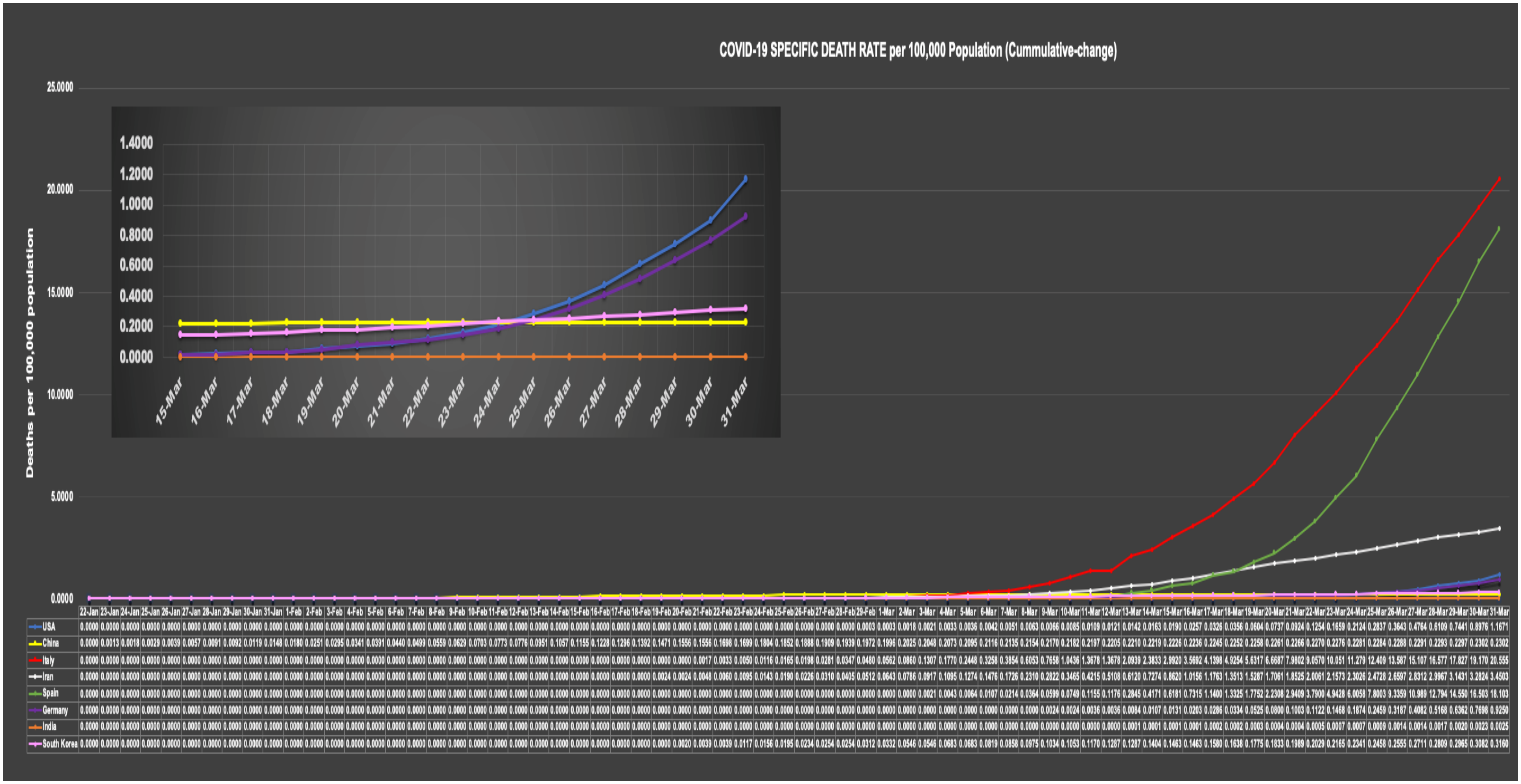

	22-Jan	23-Jan	24-Jan	25-Jan	26-Jan	27-Jan	28-Jan	29-Jan	30-Jan	31-Jan	1-Feb	2-Feb	3-Feb	4-Feb	5-Feb	6-Feb	7-Feb	8-Feb	9-Feb	10-Feb	11-Feb	12-Feb	13-Feb	14-Feb
USA	0.0000	0.0000	0.0000	0.0000	0.0000	0.0000	0.0000	0.0000	0.0000	0.0000	0.0000	0.0000	0.0000	0.0000	0.0000	0.0000	0.0000	0.0000	0.0000	0.0000	0.0000	0.0000	0.0000	0.0000
China	0.0000	0.0013	0.0018	0.0029	0.0039	0.0057	0.0091	0.0092	0.0119	0.0148	0.0180	0.0251	0.0295	0.0341	0.0391	0.0440	0.0499	0.0559	0.0629	0.0703	0.0773	0.0776	0.0951	0.1057
Italy	0.0000	0.0000	0.0000	0.0000	0.0000	0.0000	0.0000	0.0000	0.0000	0.0000	0.0000	0.0000	0.0000	0.0000	0.0000	0.0000	0.0000	0.0000	0.0000	0.0000	0.0000	0.0000	0.0000	0.0000
Iran	0.0000	0.0000	0.0000	0.0000	0.0000	0.0000	0.0000	0.0000	0.0000	0.0000	0.0000	0.0000	0.0000	0.0000	0.0000	0.0000	0.0000	0.0000	0.0000	0.0000	0.0000	0.0000	0.0000	0.0000
Spain	0.0000	0.0000	0.0000	0.0000	0.0000	0.0000	0.0000	0.0000	0.0000	0.0000	0.0000	0.0000	0.0000	0.0000	0.0000	0.0000	0.0000	0.0000	0.0000	0.0000	0.0000	0.0000	0.0000	0.0000
Germany	0.0000	0.0000	0.0000	0.0000	0.0000	0.0000	0.0000	0.0000	0.0000	0.0000	0.0000	0.0000	0.0000	0.0000	0.0000	0.0000	0.0000	0.0000	0.0000	0.0000	0.0000	0.0000	0.0000	0.0000
India	0.0000	0.0000	0.0000	0.0000	0.0000	0.0000	0.0000	0.0000	0.0000	0.0000	0.0000	0.0000	0.0000	0.0000	0.0000	0.0000	0.0000	0.0000	0.0000	0.0000	0.0000	0.0000	0.0000	0.0000
South Korea	0.0000	0.0000	0.0000	0.0000	0.0000	0.0000	0.0000	0.0000	0.0000	0.0000	0.0000	0.0000	0.0000	0.0000	0.0000	0.0000	0.0000	0.0000	0.0000	0.0000	0.0000	0.0000	0.0000	0.0000

	15-Feb	16-Feb	17-Feb	18-Feb	19-Feb	20-Feb	21-Feb	22-Feb	23-Feb	24-Feb	25-Feb	26-Feb	27-Feb	28-Feb	29-Feb	1-Mar	2-Mar	3-Mar	4-Mar	5-Mar	6-Mar	7-Mar	8-Mar	9-Mar	10-Mar
USA	0.0000	0.0000	0.0000	0.0000	0.0000	0.0000	0.0000	0.0000	0.0000	0.0000	0.0000	0.0000	0.0000	0.0000	0.0003	0.0003	0.0018	0.0021	0.0033	0.0036	0.0042	0.0051	0.0063	0.0066	0.0085
China	0.1155	0.1228	0.1296	0.1392	0.1471	0.1556	0.1556	0.1698	0.1699	0.1804	0.1852	0.1888	0.1909	0.1939	0.1972	0.1996	0.2025	0.2048	0.2073	0.2095	0.2116	0.2135	0.2154	0.2170	0.2182
Italy	0.0000	0.0000	0.0000	0.0000	0.0000	0.0000	0.0017	0.0033	0.0050	0.0116	0.0165	0.0198	0.0281	0.0347	0.0480	0.0562	0.0860	0.1307	0.1770	0.2448	0.3258	0.3854	0.6053	0.7658	1.0436
Iran	0.0000	0.0000	0.0000	0.0000	0.0024	0.0024	0.0048	0.0060	0.0095	0.0143	0.0190	0.0226	0.0310	0.0405	0.0512	0.0643	0.0786	0.0917	0.1095	0.1274	0.1476	0.1726	0.2310	0.2822	0.3465
Spain	0.0000	0.0000	0.0000	0.0000	0.0000	0.0000	0.0000	0.0000	0.0000	0.0000	0.0000	0.0000	0.0000	0.0000	0.0000	0.0000	0.0000	0.0021	0.0043	0.0064	0.0107	0.0214	0.0364	0.0599	0.0749
Germany	0.0000	0.0000	0.0000	0.0000	0.0000	0.0000	0.0000	0.0000	0.0000	0.0000	0.0000	0.0000	0.0000	0.0000	0.0000	0.0000	0.0000	0.0000	0.0000	0.0000	0.0000	0.0000	0.0000	0.0024	0.0024
India	0.0000	0.0000	0.0000	0.0000	0.0000	0.0000	0.0000	0.0000	0.0000	0.0000	0.0000	0.0000	0.0000	0.0000	0.0000	0.0000	0.0000	0.0000	0.0000	0.0000	0.0000	0.0000	0.0000	0.0000	0.0000
South Korea	0.0000	0.0000	0.0000	0.0000	0.0000	0.0020	0.0039	0.0039	0.0117	0.0156	0.0195	0.0234	0.0254	0.0254	0.0312	0.0332	0.0546	0.0546	0.0683	0.0683	0.0819	0.0858	0.0975	0.1034	0.1053

	11-Mar	12-Mar	13-Mar	14-Mar	15-Mar	16-Mar	17-Mar	18-Mar	19-Mar	20-Mar	21-Mar	22-Mar	23-Mar	24-Mar	25-Mar	26-Mar	27-Mar	28-Mar	29-Mar	30-Mar	31-Mar
USA	0.0109	0.0121	0.0142	0.0163	0.0190	0.0257	0.0326	0.0356	0.0604	0.0737	0.0924	0.1254	0.1659	0.2124	0.2837	0.3643	0.4764	0.6109	0.7441	0.8976	1.1671
China	0.2197	0.2205	0.2210	0.2219	0.2226	0.2236	0.2245	0.2252	0.2258	0.2261	0.2266	0.2270	0.2276	0.2281	0.2284	0.2288	0.2291	0.2293	0.2297	0.2302	0.2302
Italy	1.3678	1.3678	2.0939	2.3833	2.9920	3.5692	4.1398	4.9254	5.6317	6.6687	7.9802	9.0570	10.051	11.279	12.409	13.587	15.107	16.577	17.827	19.170	20.555
Iran	0.4215	0.5108	0.6120	0.7274	0.8620	1.0156	1.1763	1.3513	1.5287	1.7061	1.8525	2.0061	2.1573	2.3026	2.4728	2.6597	2.8312	2.9967	3.1431	3.2824	3.4503
Spain	0.1155	0.1176	0.2845	0.4171	0.6181	0.7315	1.1400	1.3325	1.7752	2.2308	2.9409	3.7900	4.9428	6.0058	7.8003	9.3359	10.989	12.794	14.550	16.503	18.103
Germany	0.0036	0.0036	0.0084	0.0107	0.0131	0.0203	0.0286	0.0334	0.0525	0.0800	0.1003	0.1122	0.1468	0.1874	0.2459	0.3187	0.4082	0.5168	0.6362	0.7698	0.9250
India	0.0000	0.0000	0.0001	0.0001	0.0001	0.0001	0.0002	0.0002	0.0003	0.0004	0.0004	0.0005	0.0007	0.0007	0.0009	0.0014	0.0014	0.0017	0.0020	0.0023	0.0025
South Korea	0.1170	0.1287	0.1287	0.1404	0.1463	0.1463	0.1580	0.1638	0.1775	0.1833	0.1989	0.2029	0.2165	0.2341	0.2458	0.2555	0.2711	0.2809	0.2965	0.3082	0.3160

Figure S3B: COVID-19 Specific death rate (Daily-change)

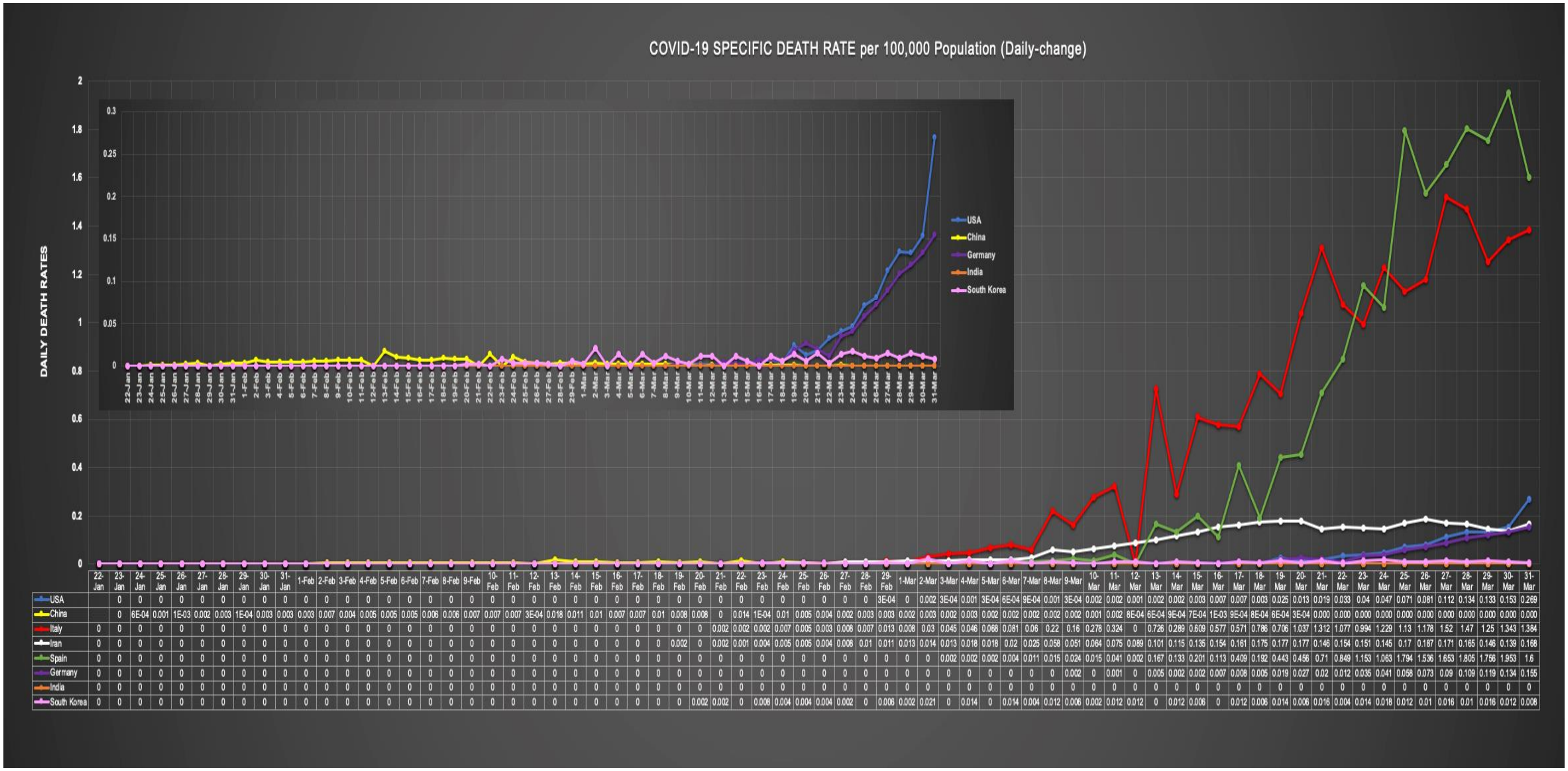

Figure S4: County-specific timeline of doubling time for cases and deaths

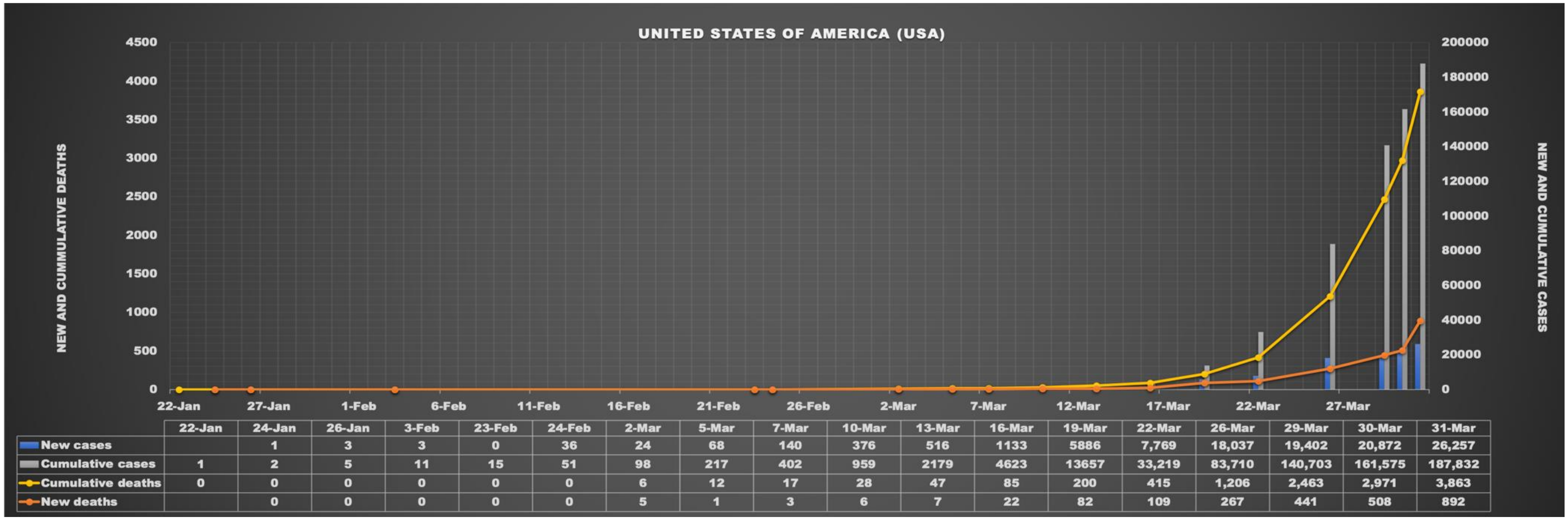

	22-Jan	24-Jan	26-Jan	3-Feb	23-Feb	24-Feb	2-Mar	5-Mar	7-Mar	10-Mar	13-Mar	16-Mar	19-Mar	22-Mar	26-Mar	29-Mar	30-Mar	31-Mar
New cases		1	3	3	0	36	24	68	140	376	516	1133	5886	7,769	18,037	19,402	20,872	26,257
Cumulative cases	1	2	5	11	15	51	98	217	402	959	2179	4623	13657	33,219	83,710	140,703	161,575	187,832
Cumulative deaths	0	0	0	0	0	0	6	12	17	28	47	85	200	415	1,206	2,463	2,971	3,863
New deaths		0	0	0	0	0	5	1	3	6	7	22	82	109	267	441	508	892

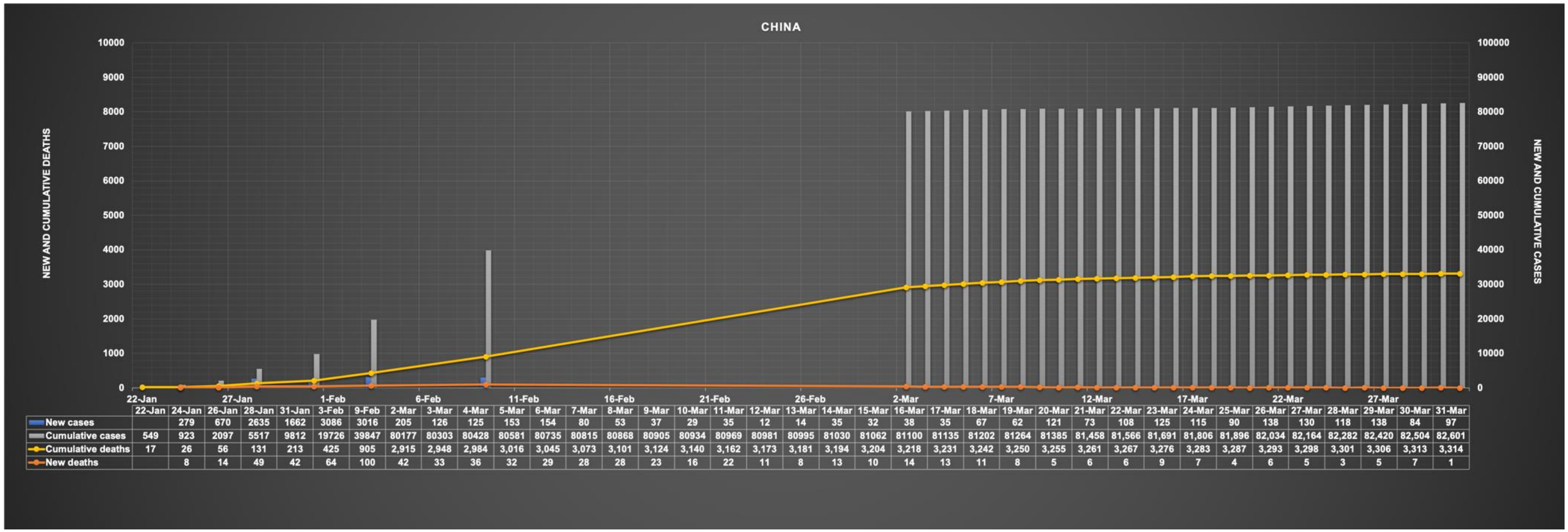

	22-Jan	24-Jan	26-Jan	28-Jan	31-Jan	3-Feb	9-Feb	2-Mar	3-Mar	4-Mar	5-Mar	6-Mar	7-Mar	8-Mar	9-Mar	10-Mar	11-Mar	12-Mar	13-Mar	14-Mar
New cases		279	670	2635	1662	3086	3016	205	126	125	153	154	80	53	37	29	35	12	14	35
Cumulative cases	549	923	2097	5517	9812	19726	39847	80177	80303	80428	80581	80735	80815	80868	80905	80934	80969	80981	80995	81030
Cumulative deaths	17	26	56	131	213	425	905	2,915	2,948	2,984	3,016	3,045	3,073	3,101	3,124	3,140	3,162	3,173	3,181	3,194
New deaths		8	14	49	42	64	100	42	33	36	32	29	28	28	23	16	22	11	8	13

	15-Mar	16-Mar	17-Mar	18-Mar	19-Mar	20-Mar	21-Mar	22-Mar	23-Mar	24-Mar	25-Mar	26-Mar	27-Mar	28-Mar	29-Mar	30-Mar	31-Mar
New cases	32	38	35	67	62	121	73	108	125	115	90	138	130	118	138	84	97
Cumulative cases	81062	81100	81135	81202	81264	81385	81,458	81,566	81,691	81,806	81,896	82,034	82,164	82,282	82,420	82,504	82,601
Cumulative deaths	3,204	3,218	3,231	3,242	3,250	3,255	3,261	3,267	3,276	3,283	3,287	3,293	3,298	3,301	3,306	3,313	3,314
New deaths	10	14	13	11	8	5	6	6	9	7	4	6	5	3	5	7	1

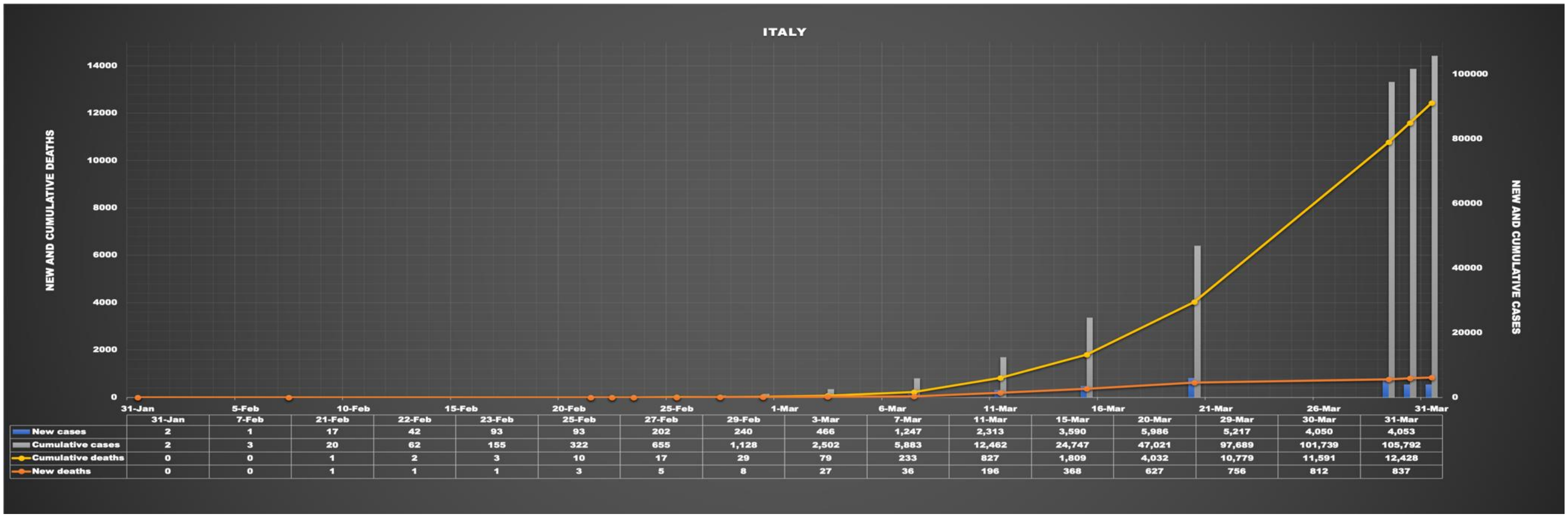

	31-Jan	7-Feb	21-Feb	22-Feb	23-Feb	25-Feb	27-Feb	29-Feb	3-Mar	7-Mar	11-Mar	15-Mar	20-Mar	29-Mar	30-Mar	31-Mar
New cases	2	1	17	42	93	93	202	240	466	1,247	2,313	3,590	5,986	5,217	4,050	4,053
Cumulative cases	2	3	20	62	155	322	655	1,128	2,502	5,883	12,462	24,747	47,021	97,689	101,739	105,792
Cumulative deaths	0	0	1	2	3	10	17	29	79	233	827	1,809	4,032	10,779	11,591	12,428
New deaths	0	0	1	1	1	3	5	8	27	36	196	368	627	756	812	837

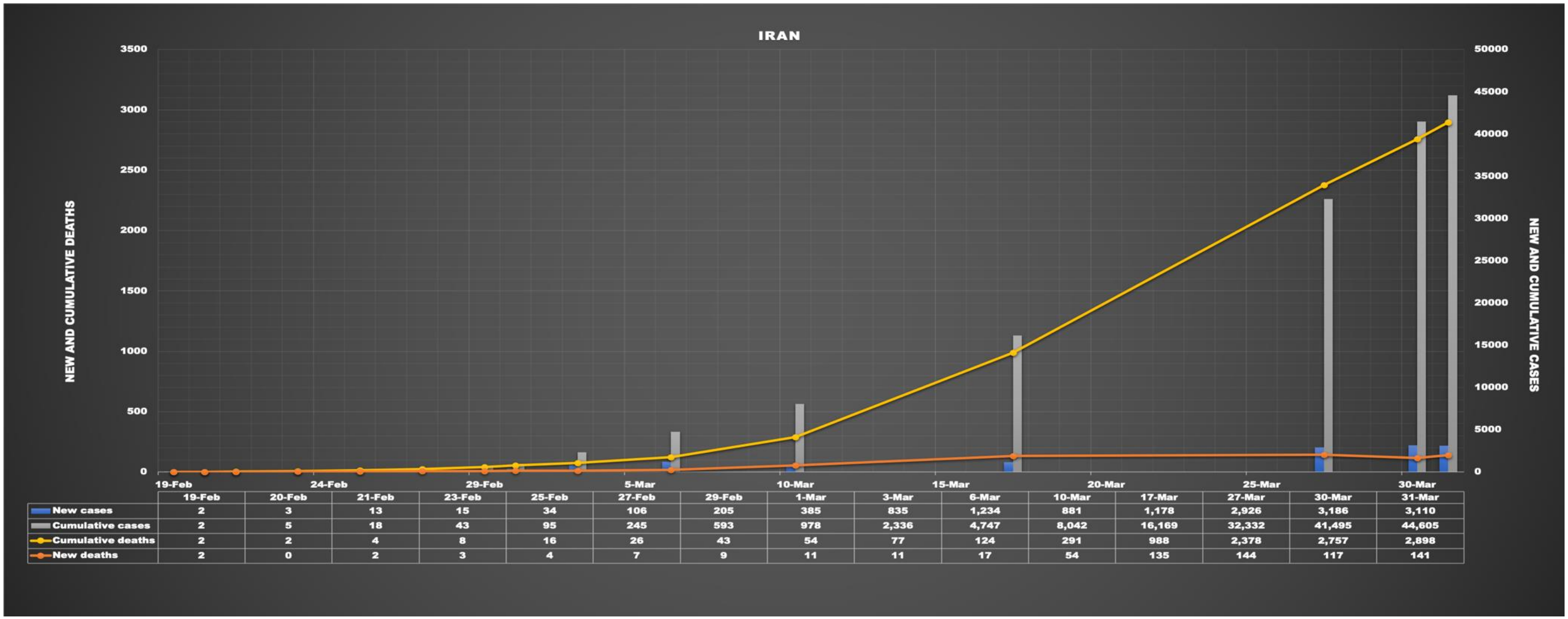

	19-Feb	20-Feb	21-Feb	23-Feb	25-Feb	27-Feb	29-Feb	1-Mar	3-Mar	6-Mar	10-Mar	17-Mar	27-Mar	30-Mar	31-Mar
New cases	2	3	13	15	34	106	205	385	835	1,234	881	1,178	2,926	3,186	3,110
Cumulative cases	2	5	18	43	95	245	593	978	2,336	4,747	8,042	16,169	32,332	41,495	44,605
Cumulative deaths	2	2	4	8	16	26	43	54	77	124	291	988	2,378	2,757	2,898
New deaths	2	0	2	3	4	7	9	11	11	17	54	135	144	117	141

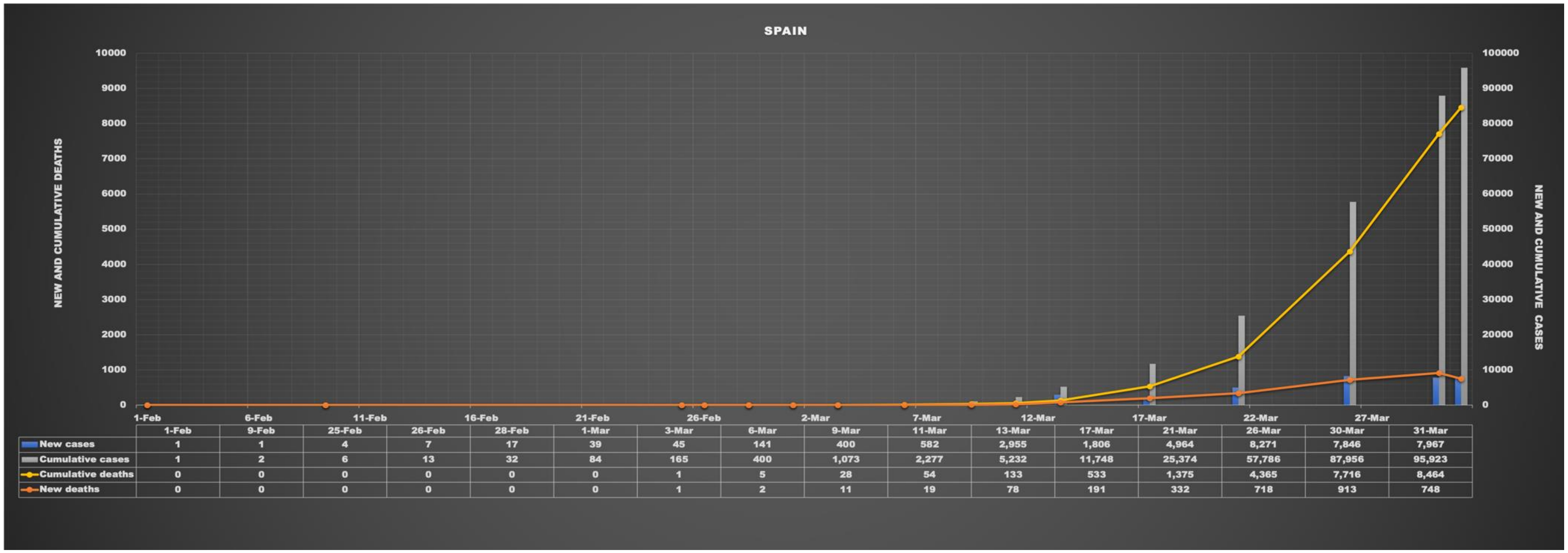

	1-Feb	9-Feb	25-Feb	26-Feb	28-Feb	1-Mar	3-Mar	6-Mar	9-Mar	11-Mar	13-Mar	17-Mar	21-Mar	26-Mar	30-Mar	31-Mar
New cases	1	1	4	7	17	39	45	141	400	582	2,955	1,806	4,964	8,271	7,846	7,967
Cumulative cases	1	2	6	13	32	84	165	400	1,073	2,277	5,232	11,748	25,374	57,786	87,956	95,923
Cumulative deaths	0	0	0	0	0	0	1	5	28	54	133	533	1,375	4,365	7,716	8,464
New deaths	0	0	0	0	0	0	1	2	11	19	78	191	332	718	913	748

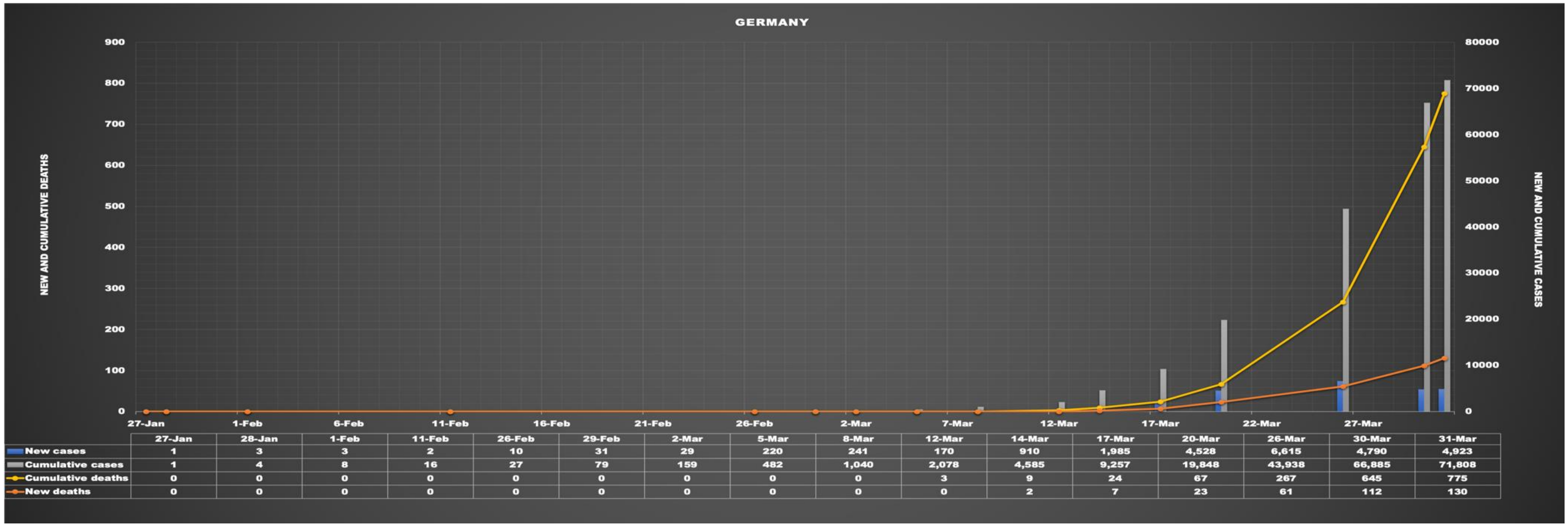

	27-Jan	28-Jan	1-Feb	11-Feb	26-Feb	29-Feb	2-Mar	5-Mar	8-Mar	12-Mar	14-Mar	17-Mar	20-Mar	26-Mar	30-Mar	31-Mar
New cases	1	3	3	2	10	31	29	220	241	170	910	1,985	4,528	6,615	4,790	4,923
Cumulative cases	1	4	8	16	27	79	159	482	1,040	2,078	4,585	9,257	19,848	43,938	66,885	71,808
Cumulative deaths	0	0	0	0	0	0	0	0	0	3	9	24	67	267	645	775
New deaths	0	0	0	0	0	0	0	0	0	0	2	7	23	61	112	130

INDIA

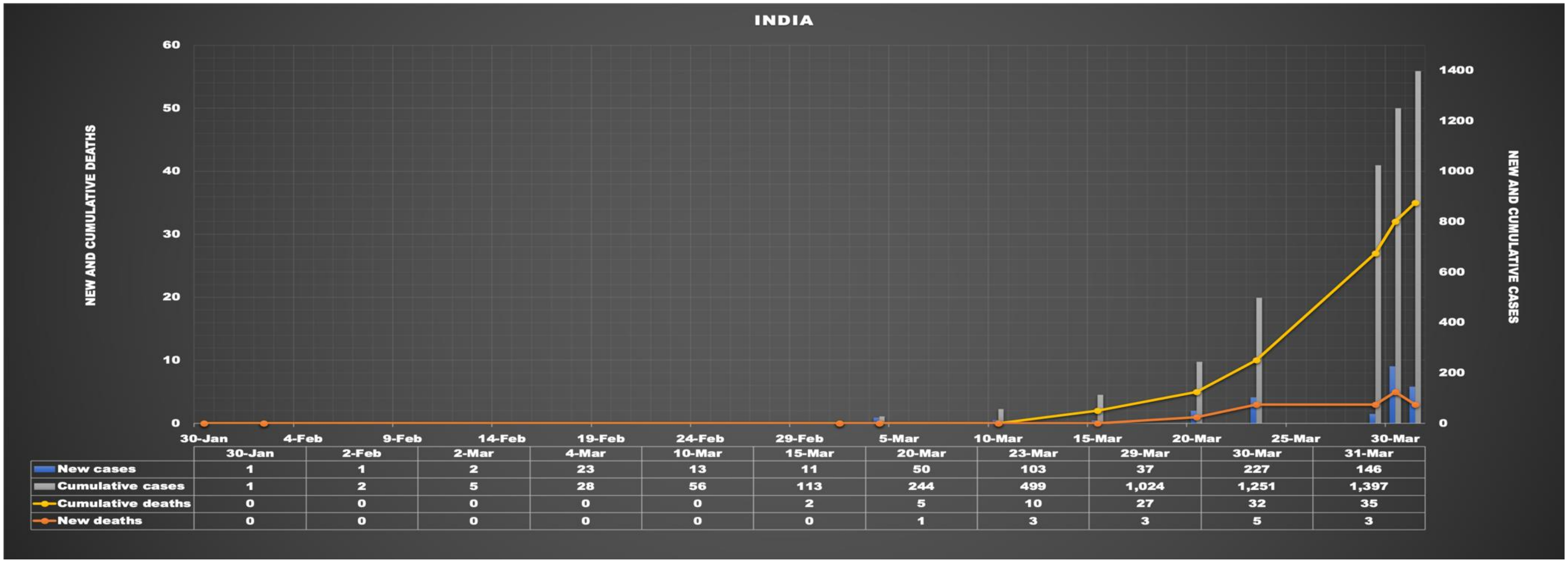

	30-Jan	2-Feb	2-Mar	4-Mar	10-Mar	15-Mar	20-Mar	23-Mar	29-Mar	30-Mar	31-Mar
New cases	1	1	2	23	13	11	50	103	37	227	146
Cumulative cases	1	2	5	28	56	113	244	499	1,024	1,251	1,397
Cumulative deaths	0	0	0	0	0	2	5	10	27	32	35
New deaths	0	0	0	0	0	0	1	3	3	5	3

SOUTH KOREA

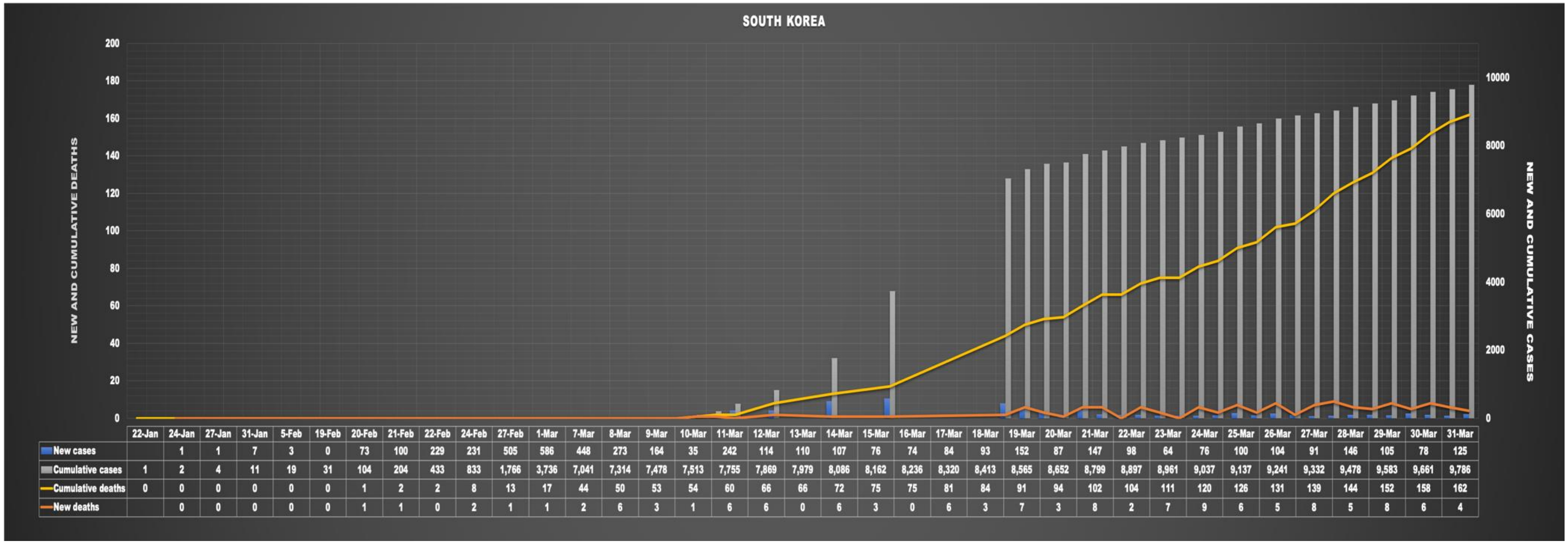

	22-Jan	24-Jan	27-Jan	31-Jan	5-Feb	19-Feb	20-Feb	21-Feb	22-Feb	24-Feb	27-Feb	1-Mar	7-Mar	8-Mar	9-Mar	10-Mar	11-Mar	12-Mar	13-Mar
New cases		1	1	7	3	0	73	100	229	231	505	586	448	273	164	35	242	114	110
Cumulative cases	1	2	4	11	19	31	104	204	433	833	1,766	3,736	7,041	7,314	7,478	7,513	7,755	7,869	7,979
Cumulative deaths	0	0	0	0	0	0	1	2	2	8	13	17	44	50	53	54	60	66	66
New deaths		0	0	0	0	0	1	1	0	2	1	1	2	6	3	1	6	6	0

	14-Mar	15-Mar	16-Mar	17-Mar	18-Mar	19-Mar	20-Mar	21-Mar	22-Mar	23-Mar	24-Mar	25-Mar	26-Mar	27-Mar	28-Mar	29-Mar	30-Mar	31-Mar
New cases	107	76	74	84	93	152	87	147	98	64	76	100	104	91	146	105	78	125
Cumulative cases	8,086	8,162	8,236	8,320	8,413	8,565	8,652	8,799	8,897	8,961	9,037	9,137	9,241	9,332	9,478	9,583	9,661	9,786
Cumulative deaths	72	75	75	81	84	91	94	102	104	111	120	126	131	139	144	152	158	162
New deaths	6	3	0	6	3	7	3	8	2	7	9	6	5	8	5	8	6	4
